# Retraction Note: Palmitoyl transferases act as novel drug targets for pancreatic cancer

**DOI:** 10.1186/s12967-024-05202-x

**Published:** 2024-04-24

**Authors:** Zhiqing Lin, Ziru Lv, Xin Liu, Keke Huang

**Affiliations:** 1 Department of Ophthalmology, The Third People’s Hospital of Chengdu, The Affiliated Hospital of Southwest Jiaotong University, 610031 Chengdu, China; 2 School of Ophthalmology and Optometry, Eye Hospital, Wenzhou Medical University, 325027 Wenzhou, China

**Retraction Note: Journal of Translational Medicine (2023) 21:249**

10.1186/s12967-023-04098-3

The authors have retracted this article because they do not have ownership of the data reported. Zhiqing Lin, Ziru Lv and Keke Huang agree with this retraction. Xin Liu has not responded to correspondence from the Publisher about this retraction.

